# Network analysis and the impact of Aflibercept on specific mediators of angiogenesis in HUVEC cells

**DOI:** 10.1111/jcmm.16778

**Published:** 2021-07-11

**Authors:** Hamid Latifi‐Navid, Zahra‐Soheila Soheili, Shahram Samiei, Mehdi Sadeghi, Sepideh Taghizadeh, Ehsan Ranaei Pirmardan, Hamid Ahmadieh

**Affiliations:** ^1^ Department of Molecular Medicine National Institute of Genetic Engineering and Biotechnology Tehran Iran; ^2^ Blood Transfusion Research Center High Institute for Research and Education in Transfusion Medicine Tehran Iran; ^3^ Department of Medical Genetics National Institute for Genetic Engineering and Biotechnology Tehran Iran; ^4^ School of Biological Sciences Institute for Research in Fundamental Sciences (IPM) Tehran Iran; ^5^ Ocular Tissue Engineering Research Center Molecular Biomarkers Nano‐Imaging Laboratory Brigham and Women's Hospital Boston Massachusetts USA; ^6^ Department of Radiology Harvard Medical School Boston Massachusetts USA; ^7^ Ophthalmic Research Center, Research Institute for Ophthalmology and Vision Science Shahid Beheshti University of Medical Sciences Tehran Iran

**Keywords:** aflibercept, angiogenesis, HUVEC cells, inflammation, matrix proteins, network analysis

## Abstract

Angiogenesis, inflammation and endothelial cells’ migration and proliferation exert fundamental roles in different diseases. However, more studies are needed to identify key proteins and pathways involved in these processes. Aflibercept has received the approval of the US Food and Drug Administration (FDA) for the treatment of wet AMD and colorectal cancer. Moreover, the effect of Aflibercept on VEGFR2 downstream signalling pathways has not been investigated yet. Here, we integrated text mining data, protein‐protein interaction networks and multi‐experiment microarray data to specify candidate genes that are involved in VEGFA/VEGFR2 signalling pathways. Network analysis of candidate genes determined the importance of the nominated genes via different centrality parameters. Thereupon, several genes—with the highest centrality indexes—were recruited to investigate the impact of Aflibercept on their expression pattern in HUVEC cells. Real‐time PCR was performed, and relative expression of the specific genes revealed that Aflibercept modulated angiogenic process by VEGF/PI3KA/AKT/mTOR axis, invasion by MMP14/MMP9 axis and inflammation‐related angiogenesis by IL‐6‐STAT3 axis. Data showed Aflibercept simultaneously affected these processes and determined the nominated axes that had been affected by the drug. Furthermore, integrating the results of Aflibercept on expression of candidate genes with the current network analysis suggested that resistance against the Aflibercept effect is a plausible process in HUVEC cells.

## 1 INTRODUCTION

Angiogenesis, process of the formation of new blood vessels from pre‐existing ones, has a fundamental role in physiological conditions and different diseases.^1^ VEGFA is the main key driver of angiogenesis signalling pathway and has different functions in this process. Binding VEGFA to VEGFR2 seems to mediate several roles including induction of angiogenesis and proliferation of endothelial cells.^1^ Therefore, blockade of the VEGFA‐VEGFR2 signalling pathway is an important key target for developing an anti‐angiogenic therapeutic system.^2^ There are several mechanisms for developing VEGF (signalling pathway)‐targeted agents that contain VEGF neutralizing antibodies (eg Aflibercept (Eylea)), tyrosine kinase inhibitors (eg Sorafenib) and antibodies that inhibit signalling pathway through binding to VEGFRs (Ramucirumab).^2^ Binding VEGFA to its receptor (VEGFR2) leads to the initiation of different downstream signalling pathways such as PI3K‐AKT‐mTOR module, Ras/Raf/MEK/ERK signalling cascade, PLC‐PKC, Rhoa, STAT, NF‐κB, JNK, FAK and RAK modules. Moreover, there are some cytokines and matrix proteins involved in the angiogenic process.^3^ Although the role of angiogenesis, inflammation and matrix‐related proteins in different diseases is well established by prior studies, little attention has been paid to determine the multiple key proteins, important molecular pathways and interactions between them via system biology approaches. Aflibercept has received the approval of the US Food and Drug Administration (FDA) for the treatment of wet AMD and colorectal cancer.^4^, ^5^ It was recruited in the study since it is a novel recombinant fusion protein that acts as a soluble decoy receptor and could bind to all isoforms of VEGFA, VEGFB and placental growth factor (PLGF).^6^ Although the anti‐VEGF effect of Aflibercept in pathologic angiogenesis is quietly clear, its possible role in physiologic angiogenesis state, components of VEGFR2‐dependent downstream signalling pathways, cytokines and matrix protein have not been investigated yet.

Protein‐protein interaction (PPI) network is one of the important tools for a comprehensive investigation of complicated biological processes in living cells. Identification of the key nodes via network analysis with topological features such as degree centrality, closeness, betweenness, centroid value, bridging, eccentricity and eigenvector centrality index enables us to determine novel therapeutic targets related to specific diseases.^7^ Degree centrality is the most commonly used local parameter for identifying the regulatory importance node based on the number of edges connected to it. Highly connected nodes interact with multiple proteins and play a fundamental regulatory role in a wide range of biological activities such as signalling module coordination, amplification and gene expression pathways.^8^, ^9^, ^10^, ^11^ However, it is well established that the nodes with low connectivity could also mediate important functions in the protein‐protein interaction network.^12^, ^13^, ^14^, ^15^, ^16^, ^17^ This is due to the fundamental importance of these nodes in other centrality parameters which are involved in topological network analysis. Betweenness centrality is used to determine key nodes in the maintenance of the functionality and coherence of biological networks. This centrality is determined based on the shortest paths that are used to delineate the number of times that a distinct node is applied to keep distant proteins connected. Therefore, betweenness centrality identifies key nodes that are observed with a high proportion in paths, between other nodes of the network.^13^, ^18^, ^19^, ^20^, ^21^ Centroid values play a fundamental role in orchestrating the activity of distinct protein clusters. Indeed, coordination of highly connected proteins and organizing functional units are performed by nodes with highly centroid values.^22^ The probability of functional association of one protein with others in the biological network evaluates by the reciprocal of the sum of the geodesic distances of a specific node to the entire network. This feature is called closeness centrality. Nodes with high closeness centrality are showing critical regulatory effect on other proteins. Moreover, any changes in the network are more likely affecting these types of proteins.^20^, ^23^ The identification of specific nodes which are easily reachable by other proteins occurs by reciprocal of the maximum of shortest path lengths. This concept is called eccentricity centrality. As a consequence, nodes with high eccentricity are easily affected or exposed to other proteins.^20^, ^24^ Bridging centrality consists of the betweenness centrality and bridging coefficient. Nodes with high bridging coefficient have highly connected first neighbours. As a result, bridging centrality is used to determine nodes that link (due to betweenness centrality components) clusters or densely connected regions (due to bridging coefficient component).^13^ Eigenvector centrality is used to distinguish central super regulatory nodes in biological networks. These nodes represent key targets in gene regulatory pathways. Nodes with high eigenvector centrality are identified by their position and the neighbouring nodes.^20^, ^25^, ^26^, ^27^ Despite the distinct importance of each centrality in the biological network, for more accurate identification of the crucial proteins, all results of centralities should be integrated without any preferences. It is important to consider that high score protein in multiple centrality parameters represents great importance in the functionality of a biological network. Our ontology analysis results demonstrated that tumour necrosis factor‐alpha (TNF‐α) signalling pathway is the most enriched pathway related to this interrelation network.

Tumour necrosis factor‐alpha (TNF‐α) and its receptors (TNFRs) trigger several signalling pathways that regulate different cellular functions such as inflammatory gene expression, cell proliferation and programmed cell death.^28^ Also, regulation of regulatory T cells (Tregs) function, endothelial cell adhesion and permeability and accumulation of immune cells including lymphocytes and monocytes to regions of inflammation was performed by TNF signalling pathway.^29^

Here, we integrated text mining data,^3^ angiogenesis‐related protein‐protein interaction networks ^30^, ^31^ and multi‐experiment microarray data ^32^, ^33^, ^34^ to find out candidate genes involved in VEGFA/VEGFR2 signalling pathways. We then aimed to do topological analysis of candidate genes’ biological networks to determine selected genes and to search for in vitro effects of Aflibercept on expression of selected genes in endothelial cells. Different kinds of endothelial cells such as SVEC4‐10 (mouse), 3B‐11 (mouse) and HUVEC (human) cells could be evaluated in the assessment. We selected the HUVECs (human umbilical vein endothelial cells). Anyway, the routine method for tube formation in the context of all the three cell types is the same.^35^

HUVECs have been extensively used as a primary, non‐immortalized cell model to study how different manipulations and pro‐ and anti‐angiogenic compounds affect endothelial cells’ migration and proliferation, and how this regulates the formation of blood vessels.^36^ Although studies in HUVECs do not represent all endothelial cell types found in an organism, HUVECs are an excellent model for the study of vascular endothelium properties and the main biological pathways that are involved in endothelium function.^37^

It has been previously established that, in endothelial cells, endogenous VEGFA forms complex with VEGFR2 and leads to trigger downstream signalling pathway. Endogenous VEGFA also controls expression of VEGFR2, vascular endothelial cadherin (VE‐cadherin) and Tie2 protein. A 44‐bp transcriptional enhancer sequence plays a prominent role in the transcription regulation of VEGFR2 by the endogenous VEGFA protein. This regulation is performed by the binding FOX:ETS motif to the FoxC2 and Ets transcription factors.^38^, ^39^ Therefore, neutralization of endogenous VEGFA seems to be convenient for investigation of VEGFR2 downstream signalling pathways.^38^

The candidate genes involved in this study were classified into three different sections: angiogenesis‐related genes (PIK3CA, PIK3R1, mTOR, AKT1, ANGPT2, STAT3, VEGFC, MAPK1, BCL2L1, RCAN1, NR4A1, CDKN1B, CCND1, ARHGAP22, PLCβ3, FOXOI, ACKR3, PTGS2, PLAU, CTNNB1), inflammation‐related genes (IL‐6, CCL2, TNFAIP6, CXCL1, C3, C5, CFB and CFI) and matrix‐related genes (MMP2, MMP9, MMP10, MMP14, ADAMTS1 and ADAMTS5).

## 2 MATERIALS AND METHODS

### 2.1 Angiogenesis, inflammation and matrix protein‐related genes (data sources)

**FIGURE 1:**
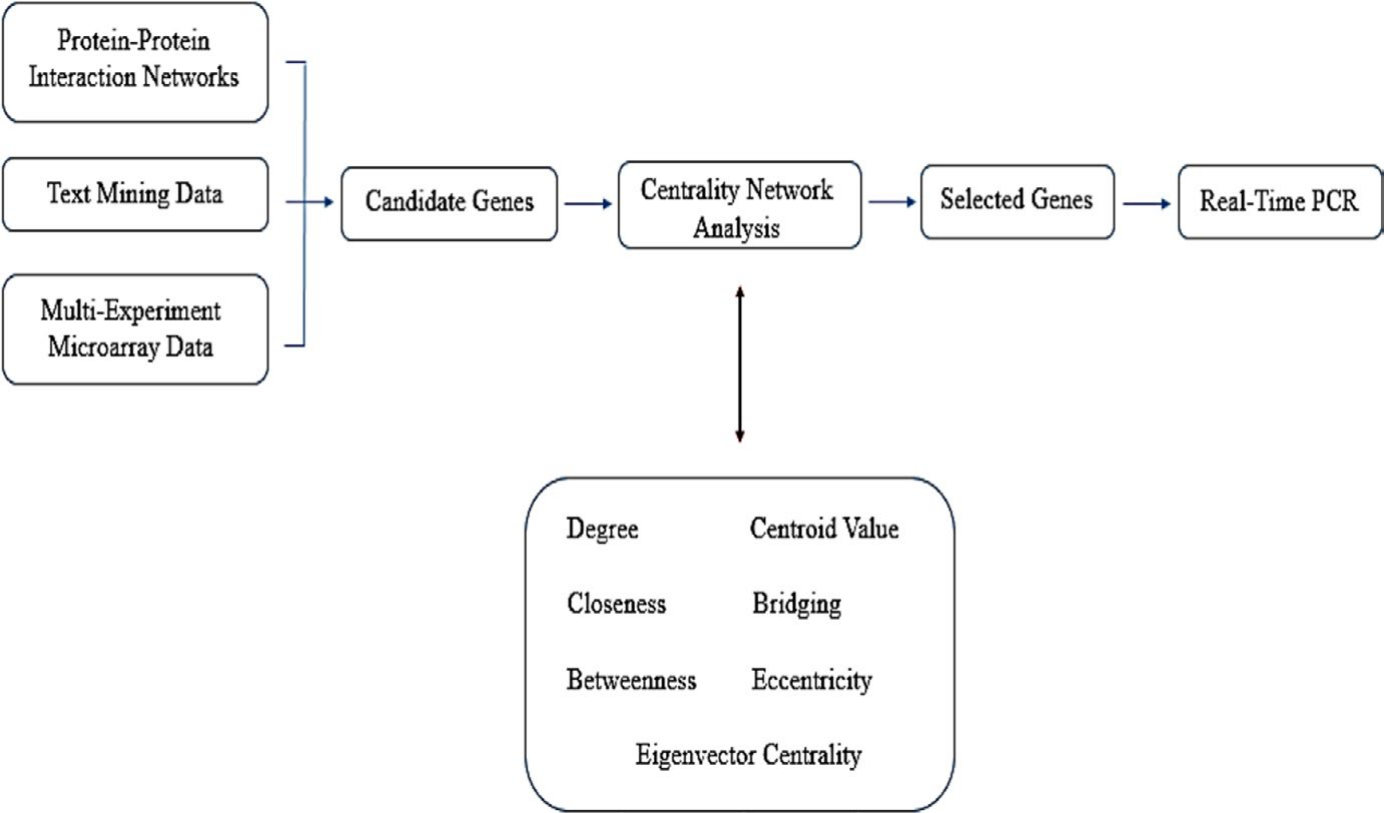
A schematic view of the steps performed in this project

A literature review from different kinds of studies, which included text mining, microarray data results and protein‐protein interaction networks, was performed to obtain the information not only associated with physiological angiogenesis and VEGFA/VEGFR2 downstream signalling pathway‐related proteins but also on the inflammatory and matrix proteins with important roles in angiogenesis. This process was carried out by using the keywords: angiogenesis, VEGFA, vascular endothelial growth factor, angiogenesis signalling pathways, angiogenesis signalling network, MMP, matrix metalloproteinase, VEGFR2 and inflammation. The results were combined, and proteins were selected with the highest repetition frequencies in all kinds of studies and then defined as the seed proteins (Table 1). Figure 1 represents a schematic view of the steps performed in the project.

**TABLE 1:** Seed proteins

Gene names	Description	Gene names	Description
MMP2	Matrix metallopeptidase 9	CXCL1	C‐X‐C motif chemokine ligand 1
MMP9	Matrix metallopeptidase 2	ADAMTS5	ADAM metallopeptidase with thrombospondin type 5
MMP10	Matrix metallopeptidase 10	PIK3R1	phosphoinositide‐3‐kinase regulatory subunit 1
MMP14	Matrix metallopeptidase 14	PLCβ3	PLCB3 phospholipase C beta 3
C3	Complement component 3	CDKN1B	Cyclin‐dependent kinase inhibitor 1B
C5	Complement component 5	mTOR	mechanistic target of rapamycin
CFB	Complement factor B	AKT1	AKT serine/threonine kinase 1
CFI	Complement factor I	PTGS2	Prostaglandin‐endoperoxide synthase 2
CCL2	C‐C motif chemokine ligand 2	NR4A1	Nuclear receptor subfamily 4 group A member 1
BCL2L1	BCL2‐like 1	PIK3CA	phosphatidylinositol‐4,5‐bisphosphate 3‐kinase catalytic subunit alpha
IL‐6	Interleukin 6	PLAU	PLAU plasminogen activator, urokinase
CCND1	Cyclin D1	VEGFC	Vascular endothelial growth factor C
RCAN1	Regulator of calcineurin 1	ACKR3	Atypical chemokine receptor 3
ANGPT2	Angiopoietin 2	ADAMTS1	ADAM metallopeptidase with thrombospondin type 1
TNFAIP6	TNF‐alpha–induced protein 6	STAT3	Signal transducer and activator of transcription 3
FOXO1	Forkhead boxO1	ARHGAP22	Rho GTPase activating protein 22
CTNNB1	Catenin beta 1	MAPK1	Mitogen‐activated protein kinase 1

### 2.2 Network construction

To investigate the functional interrelation of 34 candidate genes, their binding proteins and associated signalling pathways, the protein‐protein interaction network was reconstructed at Homo sapiens organism by version 3.5.1 of GeneMANIA plug‐in implemented in Cytoscape software. There are different types of evidence mode that have been represented by this plug‐in which included physical interaction, co‐expression and co‐localization. Visualization of this protein‐protein interaction network was performed by Cytoscape software (version 3.6.0).^40^ Then, we detected densely interrelated regions (clusters) in the protein‐protein interaction network by molecular complex detection (MCODE) algorithm (http://baderlab.org/Software/MCODE).^41^ The Molecular Complex Detection (MCODE) as a prominent graph clustering algorithm was applied to determine densely associated regions and visualize them in biological network. This algorithm is implemented in three steps: (a) vertex weighting by the local network density which is performed based on clustering coefficient, (b) prediction of molecular complexes based on locally dense seed protein which is assigned by the highest vertex weighted and (c) adding or removing proteins due to post‐processing.^41^ The tendency of a graph to form different clusters (molecular complexes) is determined through the clustering coefficient parameter.^20^, ^42^ The Clustering Coefficient of *i* is calculated by the equation *C_i_* = 2*n*/*k_i_* (*k_i_* − 1) which is defined as follows: (a) n is the number of edges in the neighbourhood, and (b) i is a vertex with degree deg(*i*) = k. Therefore, Ci is used to determine the proportion of the number of edges between the neighbours of i to the total possible number of this kind of edges and placed between the range 0 and 1 (0 ≤ *C_i_* ≤ 1). A clustering coefficient (*C_i_*) close to 1 indicates a high probability of cluster formation by the network.^20^ It is well established that the biological networks represent a more average clustering coefficient than random networks.^21^, ^43^ The scoring and ranking criteria of the MCODE algorithm results are summarized in the following three cases: (a) the product of the complex subgraph, (b) the density and (c) the number of vertices in the complex subgraph. More dense complex is placed in the higher rank at the results.^41^

### 2.3 Enrichment analysis

To further determine the biological concepts behind the gene list and interpretation of them, an enrichment analysis was performed by DAVID (https://david.ncifcrf.gov/)(Database for Annotation, Visualization, and Integrated Discovery) that is used for analysis of gene ontology which included biological process (BP), molecular function (MP) and cellular component (CC).^44^ Three different lists of results created independently after enrichment analysis. We considered *P* < .05 as representing statistical significance. Reduction and visualization of gene ontology results were performed by REVIGO (http://revigo.irb.hr/).^45^ For allowed similarity and semantic similarity measure parameters, "Small (0.5)" and "SimRel" were recruited, respectively. Kyoto Encyclopedia of Genes and Genomes (KEGG) enrichment analyses also were performed to obtain the most enriched pathways of the interrelation network.

### 2.4 Topological network analysis

To analyse topological feathers of the network, centrality measurement was performed by CentiScaPe (version 2.2) and Gephi (version 0.9.2) to determine nodes with great centrality or topological importance. These nodes usually called hubs and had a fundamental role in different kinds of networks. Several types of centrality were analysed such as degree, betweenness, centroid value, closeness, bridging, eccentricity and eigenvector centrality. Then, we categorized each node according to its position in these seven states and determined important targets which is crucial for the integrity of these networks.

### 2.5 HUVEC cell culture and treatment

The less than 2 and more than 10 passage number of the cells is appropriate in many functional assays such as maximum tube formation and run experiments in optimum condition. In a standard protocol, it seems that the downstream signalling pathways and molecular mechanisms that are involved in the angiogenic process and the results obtained from HUVEC cells can be generalized to other types of endothelial cells.^46^ HUVECs between the 2nd and the 6th passages were cultured in Dulbecco's modified essential medium containing L‐glutamine and sodium pyruvate (DMEM; Gibco, USA) supplemented with 10% foetal bovine serum (FBS; Gibco, USA) and penicillin‐streptomycin (Sigma‐Aldrich, USA) (64‐100 mg/L) until reaching 100% confluency. The day before treatment with Aflibercept (Bayer, Germany), HUVEC cells were seeded to 70% confluency in 21‐cm^2^ dishes. 0.45 nmol/L Aflibercept was used in in vitro experiments. The aforesaid concentration is consistent to the peak of serum concentration of Aflibercept (Cmax) in vivo and after the first intravitreal injection would mimics its systemic effect reasonably. Cmax is obtained by investigating recent anti–VEGF‐associated studies that evaluated the expression level of specific genes on vascular endothelial cells.^47^, ^48^, ^49^, ^50^, ^51^, ^52^ RNA extraction was performed from HUVEC cells that had been treated 6 hours or 24 hours by Aflibercept (Bayer, Germany) or phosphate‐buffered saline (PBS, as a control).

### 2.6 RNA preparation and RT‐qPCR

Isolation of total RNA and purification of RNA samples from genomic DNA contamination were performed by using TriPure Isolation Reagent (Roche, Germany) and Accurate Genomic DNA Removal Kit (abmgood, Canada) according to the manufacturer's instructions. The quantity and quality of RNA samples were examined by a NanoDrop Spectrometer 2000 (Thermo Fisher, USA). Real‐time PCR was performed by SuperScript™ III One‐Step RT‐PCR System with Platinum™ Taq DNA Polymerase (Invitrogen, USA) according to the following program: 5 minutes at 60℃ (first step) and 5 minutes at 95℃ (second step) for cDNA synthesis and pre‐denaturation, 20 seconds at 95℃, 35 seconds at 60℃ for 45 cycles and temperature range extends from 72℃ to 95℃ for melting curve analysis. Oligonucleotide primer sequences which used for quantitative real‐time PCR have been presented in Table 2. All primers were designated by allele ID (version 7) and further improved by mfold and SnapGene (version 3.2.1) software. Calculation of relative mRNA expression was performed by the 2^∆∆Ct^ method, and the results were normalized with GAPDH as a reference gene.

**TABLE 2:** Primer list

Gene name	Forward Primer	Reverse Primer
GAPDH	GCACCACCAACTGCTTAGC	GGCATGGACTGTGGTCATGA
MMP14	GAGCTCAGGGCAGTGGATAG	GGTAGCCCGGTTCTACCTTC
AKT1	GCACAAACGAGGGGAGTACAT	CCTCACGTTGGTCCACATCC
PTGS2	TCCTGTGCCTGATGATTGCC	CTGATGCGTGAAGTGCTGG
ADAMTS5	GAACATCGACCAACTCTACTCCG	CAATGCCCACCGAACCATCT
IL‐6	ACTCACCTCTTCAGAACGAATTG	CCATCTTTGGAAGGTTCAGGTTG
STAT3	ATCACGCCTTCTACAGACTGC	CATCCTGGAGATTCTCTACCACT
FOXO1	TCGTCATAATCTGTCCCTACACA	CGGCTTCGGCTCTTAGCAAA
ANG2	ACCCCACTGTTGCTAAAGAAGA	CCATCCTCACGTCGCTGAATA
ERK2	TACACCAACCTCTCGTACATCG	CATGTCTGAAGCGCAGTAAGATT
MMP9	CTTTGACAGCGACAAGAAGTGG	ATGCCATTCACGTCGTCCTTAT
VEGFC	AGTTCCACCACCAAACATGC	TGAAGGGACACAACGACACA
mTOR	GGCCGACTCAGTAGCATGAA	CGGGCACTCTGCTCTTTGA
RCAN1	TTTAGCTCCCTGATTGCCTGT	AAAGGTGATGTCCTTGTCATACGT
PIK3CA	CCACGACCATCATCAGGTGAA	CCTCACGGAGGCATTCTAAAGT
C3	GGGGAGTCCCATGTACTCTATC	GGAAGTCGTGGACAGTAACAG
CFB	GCACTGGAGTACGTGTGTCC	CCCGTTCTCGAAGTCGTGTG
CFI	GGAAACGAATTGTGGGAGGAA	GTGCAGCAGTCAGAATCCAAC
MMP10	ATCCAAGAGGCATCCATACC	TCAACCTTAGGCTCAACTCC
PIK3R1	TGGACGGCGAAGTAAAGCATT	AGTGTGACATTGAGGGAGTCG
CDKN1B	GACTGATCCGTCGGACAGC	CACAGAACCGGCATTTGGG
MMP2	ATGACAGCTGCACCACTGAG	ATTTGTTGCCCAGGAAAGTGAAG

### 2.7 Statistical analysis

HUVEC cells were treated by Aflibercept in three independent experiments that had been performed in different time schedules. Assessment of the statistical differences among the experimental groups was performed by Student's *t* test. Results were presented as mean ± standard error of the mean (SEM). Quantitative real‐time PCR tests were performed on two independent duplicated samples, and a *P* value <.05 was considered statistically significant.

## 3 RESULTS

### 3.1 Networks of angiogenesis, inflammation and matrix proteins

**FIGURE 2:**
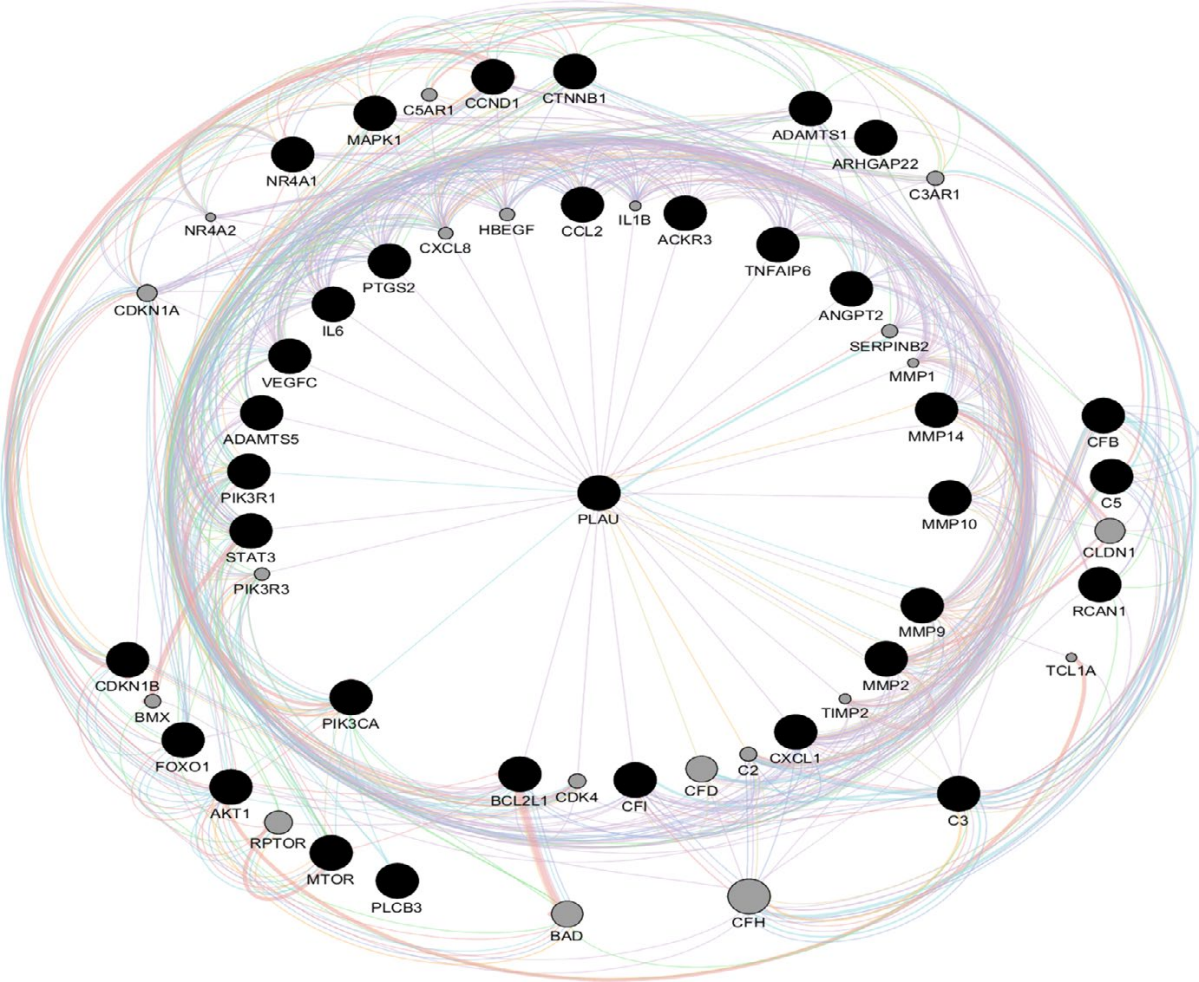
Interrelation network which included angiogenesis, inflammation and matrix‐related proteins

**FIGURE 3:**
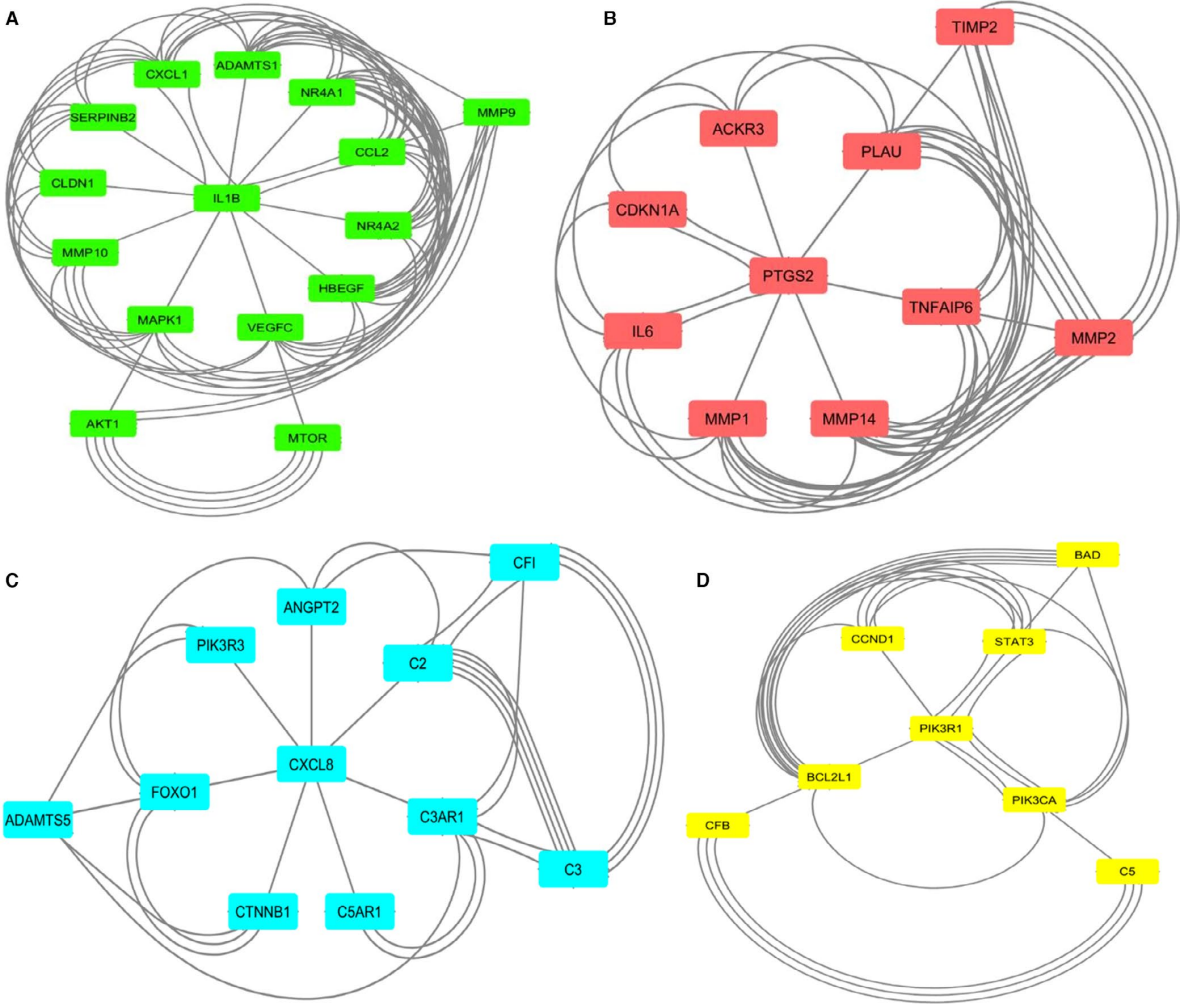
Clusters of interrelation network determined by MCODE plug‐in. (A) Cluster 1. (B) Cluster 2. (C) Cluster 3. (D) Cluster 4

Data of the specific genes of VEGFA/VEGFR2 downstream signalling pathways, inflammatory and matrix‐related proteins were obtained from text mining, microarray data results and protein‐protein interaction networks, and then, the proteins with the highest repetition in all kinds of studies (=candidate genes) were utilized to reconstruct a PPI network which included angiogenesis, inflammation and matrix‐related proteins simultaneously (Figure 2). The MCODE plug‐in implemented in Cytoscape software was applied to determine the densely interrelated regions (clusters) in the network. As shown in Figure 3, a total of four clusters were identified. There were 15, 10, 11 and 8 nodes in clusters 1, 2, 3 and 4. The obtained score was 7.429, 6.667, 4.600 and 4.571, respectively (Table 3). DAVID database was applied in each cluster to determine the most important KEGG pathways distinctly (Table 4). The results represented that Cluster 1 (with the highest score: 7.429) consists of nodes related to the TNF signalling pathway (map04668).

**TABLE 3:** MCODE clusters

MCODE Cluster	Node IDs
**1**	HBEGF, MMP9, SERPINB2, ADAMTS1, NR4A2, MMP10, IL1B, CCL2, NR4A1, AKT1, MTOR, MAPK1, CXCL1, VEGFC, CLDN1
**2**	MMP2, MMP1, MMP14, PTGS2, ACKR3, TNFAIP6, PLAU, CDKN1A, TIMP2, IL‐6
**3**	ADAMTS5, PIK3R3, C3AR1, C5AR1, C2, CTNNB1, C3, FOXO1, CXCL8, ANGPT2, CFI
**4**	PIK3R1, BCL2L1, STAT3, BAD, CCND1, PIK3CA, CFB, C5

**TABLE 4:** KEGG pathways of interrelation network and each MCODE cluster by DAVID

	Number Of nodes	KEGG pathway	Genes	*P* value
MCODE Cluster 1	15	TNF signalling pathway	HBEGF, MMP9, SERPINB2, ADAMTS1, NR4A2,MMP10, IL1B, CCL2, NR4A1, AKT1, MTOR, MAPK1, CXCL1, VEGFC, CLDN1	9.5E−7
MCODE Cluster 2	10	Pathways in cancer	MMP2, MMP1, MMP14, PTGS2, ACKR3, TNFAIP6, PLAU, CDKN1A, TIMP2, IL‐6	4.7E−4
MCODE Cluster 3	11	Staphylococcus aureus infectionComplement and coagulation cascades	ADAMTS5, PIK3R3, C3AR1, C5AR1, C2, CTNNB1, C3, FOXO1, CXCL8, ANGPT2, CFI	4.4E−7 1.6E−6
MCODE Cluster 4	8	Pancreatic cancer	PIK3R1, BCL2L1, STAT3, BAD, CCND1, PIK3CA, CFB, C5	2.3E−9

### 3.2 Enrichment analysis

**FIGURE 4:**
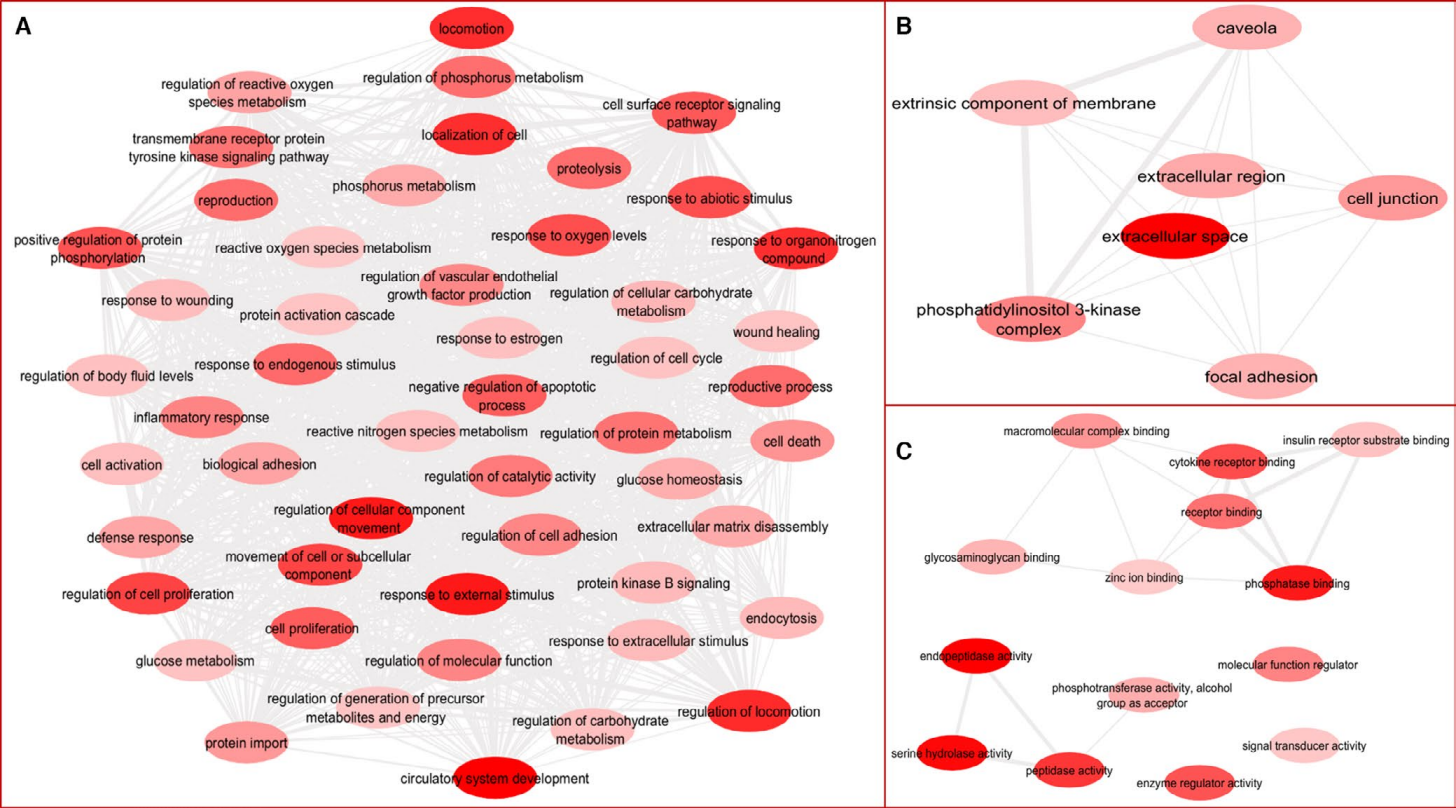
GO term networks. The P values of each GO term in this interrelation network represented by node colour (dark colour shows more abundant process). (A) Biological processes. (B) Cellular components. (C) Molecular functions

To determine the most relevant concepts behind the GO terms, enrichment analysis was performed on the interrelation network. GO terms which included biological process, molecular function and cellular component and their p values were used to reconstruct three GO networks (Figure 4). GO terms established by the DAVID tool with lower EASE score were more related and enriched with a list of gene in the network. Considering the p values, the most enriched GO terms of biological process were vasculature development (GO:0001944), cardiovascular system development F(GO:0072358), circulatory system development (GO:0072359) and blood vessel development (GO:0001568), of molecular function they were serine‐type endopeptidase activity (GO:0004252), endopeptidase activity (GO:0004175), serine‐type peptidase activity (GO:0008236), and serine hydrolase activity (GO:0017171), of cellular component they were extracellular space (GO:0005615), phosphatidylinositol 3‐kinase complex (GO:0005942), extracellular region part (GO:0044421) and cell junction (GO:0 030 054) which is displayed in dark red nodes in Figure 4 for more detail, see Tables S1 and S2.

### 3.3 Identification of hubs

**FIGURE 5:**
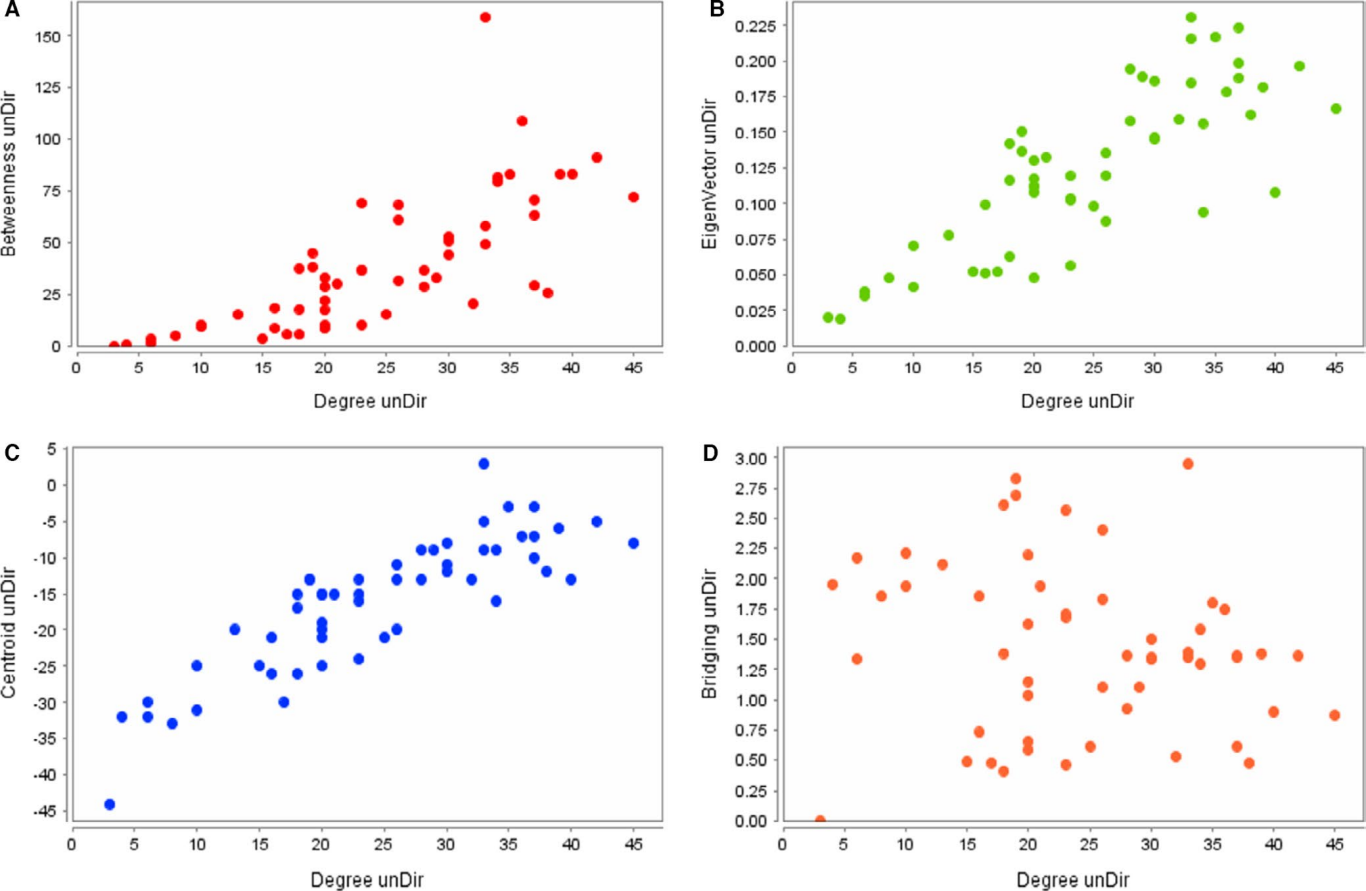
Scatter plots of the different centrality parameters. (A) Degree and betweenness centralities. (B) Degree and eigenvector centralities. (C) Degree centrality and centroid value. (D) Degree and bridging centralities

**FIGURE 6:**
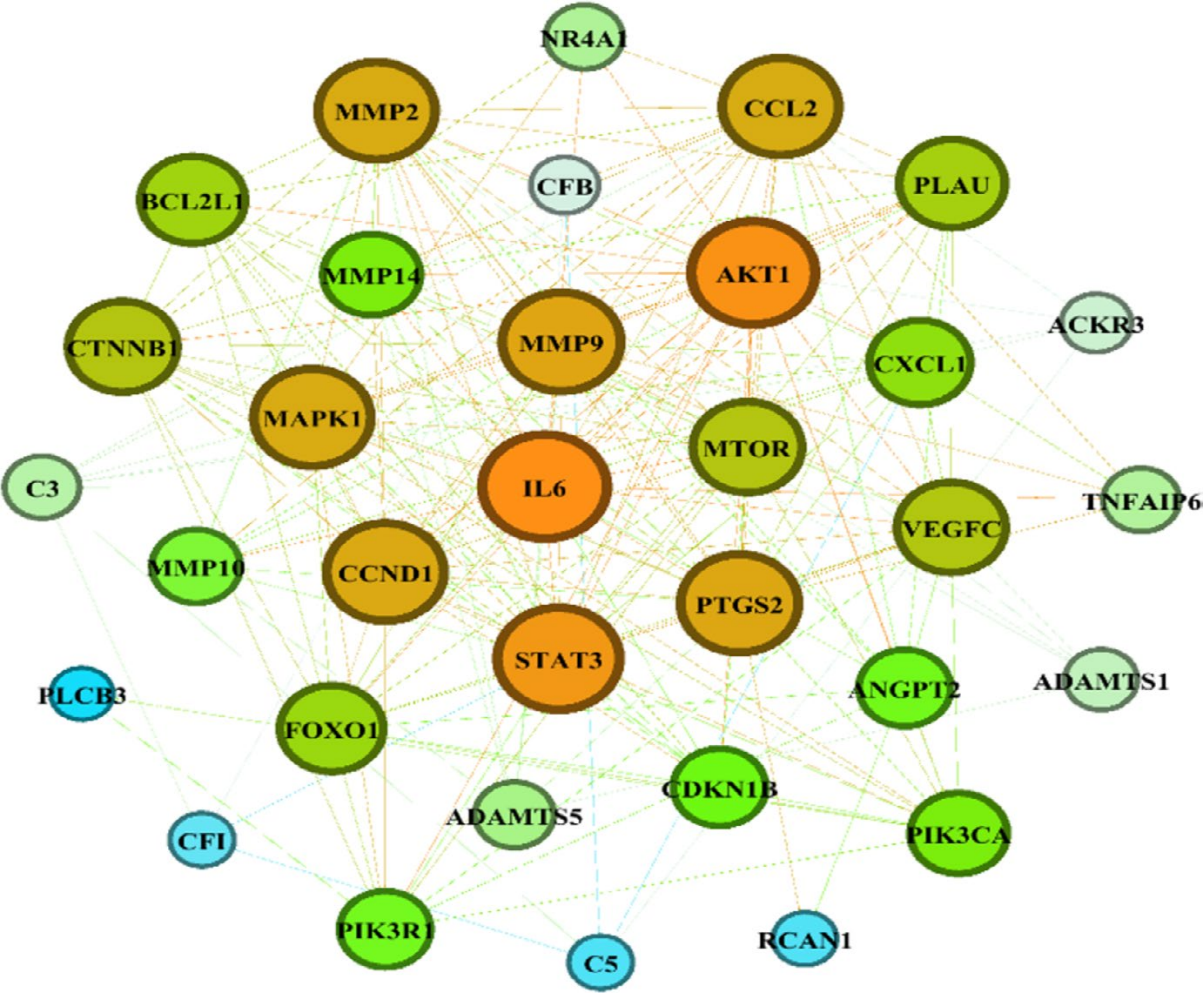
Network which represented results of eigenvector centrality analysis by string and Gephi tools

The CentiScaPe plug‐in was applied to identify the most crucial nodes in a network and determine different kinds of centrality indexes for each node. Scatter plots of nodes included highest degree with betweenness centrality, closeness centrality, centroid value, bridge node and eigenvector centrality were delineated separately, Figure 5 and Figure 6 for more detail, see Table S3, Figures S1 and S2. The results obtained from CentiScaPe were categorized from the highest to the lowest scores to determine key genes in different centralities (Table S4). To facilitate the overall assessment of distinct gene status, according to Table S4 results, we assigned ranks 1‐54 to the recruited genes by considering their order in different centralities (for more detail, see Table S5). We considered nodes that showed up to a rank about 25 as important nodes in the network. Therefore, the importance of all genes in different kinds of centrality was evaluated and the expression pattern of selected genes was investigated by qPCR in Aflibercept‐treated HUVEC cells in in vitro cultures.

### 3.4 Expression of selected genes in Aflibercept‐treated HUVEC cells

**FIGURE 7:**
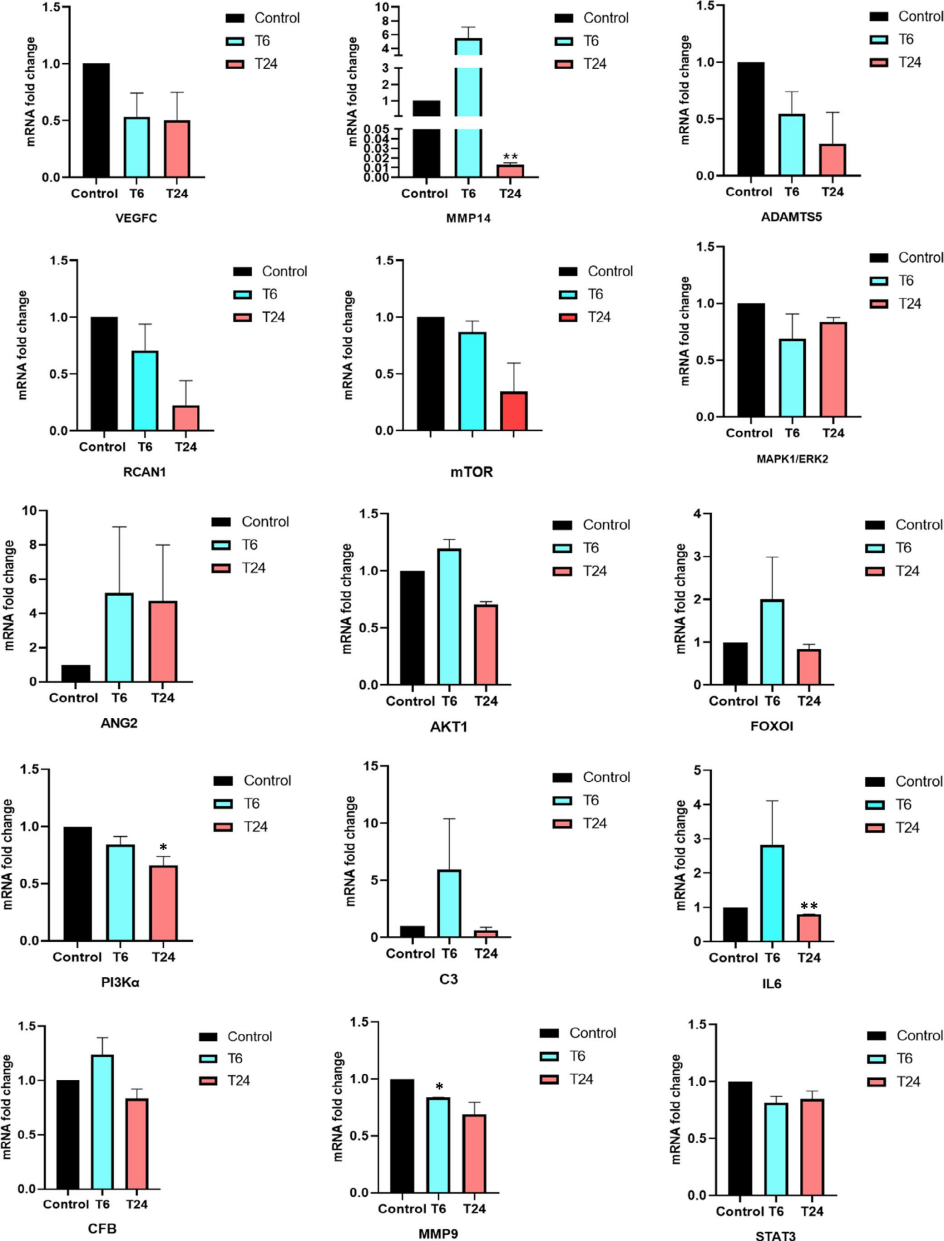
Relative expression of genes in Aflibercept‐treated HUVEC cells in in vitro cultures. Aflibercept (*C*_max_ = 0.45 nmol/L)‐treated HUVEC cells were examined for conceivable changes in relative expression levels of different angiogenesis, inflammatory and matrix‐related genes. Error bars represent means ± SE, *P < .05

The expression of nominated genes including PTGS2, MMP10, CDKN1B, ADAMTS5, C3, IL‐6, MMP2, AKT1, CFB, CFI, PIK3R1, VEGFC, MAPK1/ERK2, mTOR, MMP14, PI3Kα, RCAN1, ANG2, MMP9, FOXO I and STAT3 was examined in Aflibercept (*C*_max_ = 0.45 nmol/L)‐treated HUVEC cells by real‐time PCR analysis. Results showed that PTGS2, MMP10, CDKN1B, MMP2, CFI and PIK3R1 genes were undetectable in HUVEC cells, while variations in expression pattern of the other genes were observed in treated cultures. Relative expression of the specific genes which significantly increased or diminished 6h or 24h later in treated cultures was represented in Table 5 for more detail, see Figure 7; Tables S6 and S7.

**TABLE 5:** Gene list with significant changes in relative expression

Gene Name	6 h—Decrease (fold)	24 h—Decrease (fold)
MMP9	1.19	–
MMP14	–	76.92
PI3Kα	–	1.51
IL‐6	–	1.25

## 4 DISCUSSION

Currently, the importance of pro‐angiogenic, inflammatory and matrix‐related proteins has been confirmed in different kinds of diseases. Regarding the fundamental role of Aflibercept as a VEGF neutralizing agent, the current study aimed to investigate the possible signature of this factor on the components of VEGFR2‐dependent downstream signalling pathways, expression of crucial inflammatory factors and matrix‐related proteins. Topological analysis of the biological networks was performed and integrated into in vitro targeted study of Aflibercept recruitment in human umbilical vein endothelial cell culture and surveying for expression of the interested genes.

The most commonly used endothelial cells in angiogenesis‐related studies are HUVECs. Both VEGFR1 and VEGFR2 receptors must be present in a sufficient frequency at the cell surface of HUVEC cells when examining the effect of Aflibercept on the downstream VEGF‐VEGFR2 signalling pathway. Integrating angiogenesis‐related protein‐protein interaction network (the “angiome”) with dynamic gene expression time‐course data has revealed that VEGFR1 and VEGFR2 levels increased after 12 hours in VEGF‐treated HUVECs.^34^ Therefore, in the current study, evaluation of the effect of Aflibercept was performed 6h or 24h after treatment.

The integration of different kinds of studies was performed to identify candidate genes related to angiogenesis, inflammation and matrix‐related protein, and then, reconstruction of protein‐protein interaction networks was done to perform topological analysis and determine important genes. Although 20 genes from the entire 34 candidate genes belong to the components of the VEGF‐VEGFR2 (angiogenesis) signalling pathway, enrichment and gene ontology analysis demonstrated that the TNF (inflammation) signalling is the most significantly enriched pathway.

The role of the TNF signalling pathway in the regulation of angiogenic process is mediated by TNFR1 and TNFR2. TNFR1 activates pro‐inflammatory and cytotoxic signalling pathways while TNFR2 has a TNF receptor‐associated factor 2 (TRAF2)‐binding site and triggers alternative NF‐κB and PI3K/Akt pathways which involve in angiogenic process.^53^ Moreover, Ji et al reported that TNFR1 has a key role in TNF‐induced tumour lymphangiogenesis and metastasis by mediating the VEGFC‐VEGFR3 signalling pathway.^54^ Also, TNFR2‐Etk‐VEGFR2 complex is formed by the PI3K/Akt pathway and plays a significant role in survival, proliferation, cell adhesion and migration.^55^ Our results also cleared that the TNF signalling pathway plays a fundamental role in the regulation and functional convergence of three different processes.

To determine interfere genes in Aflibercept performance course, the network centrality analysis was performed. Distinguished genes were determined by different criteria, which included high score in all kinds of centralities (VEGFC, MAPK1, PI3KA1, MMP9, PI3KR1); in all kinds of centralities except bridging (STAT3, PTGS2); in all kinds of centralities except bridging and eccentricity (IL‐6); in centroid, degree, eigenvector and closeness centralities (MMP2, MMP14); in all kinds of centralities except degree and eccentricity (ANG2); in degree, centroid and betweenness centralities (AKT1, C3); in bridging, centroid and betweenness centralities (ADAMTS5, CFI); in bridging and centroid (CDKN1B, FOXOI, RCAN1); and in centroid index (mTOR, CFB, MMP10).

Measurements of indicator genes after in vitro recruitment of Aflibercept revealed significant changes in expression levels of PI3KA, MMP9, MMP14, IL‐6. It seemed that Aflibercept has modulated angiogenic process by VEGF/PI3KA/AKT/mTOR axis, invasion and metastasis processes by MMP14/MMP9 axis and inflammation‐related angiogenesis by IL‐6‐STAT3 signalling pathway.

Binding VEGF to its receptor VEGFR2 activates PI3K/AKT/mTOR signalling pathway which leads to numerous cellular functions such as proliferation and angiogenesis. In this research, VEGF neutralization by Aflibercept leads to a decrease in VEGF/VEGFR2 interaction, reduced the initiation of downstream PI3K/AKT/mTOR signalling pathway and down‐regulated the signalling cascade in mRNA expression or protein stability levels. mRNA expression level of the PI3KA gene was reduced after 24h in Aflibercept‐treated HUVEC cell line due to a decrease in VEGF/VEGFR2 interaction. The decrease in the amount of PI3KA gene expression, probably, leads to reduced level of conversion of PIP2 to PIP3 and reduces the progression of the angiogenic process.^56^

AKT1 and mTOR gene expression levels (unlike PI3K gene) did not show significant changes in Aflibercept‐treated HUVEC cell culture. A recent study reports that VEGF does not control AKT1 gene expression.^57^ Indeed, regulation of PKB/AKT protein stability is performed by VEGF‐controlled proteolysis.^58^ So, it seems that VEGF neutralization by Aflibercept leads to decreased PKB/AKT protein levels by increasing proteasome activity and down‐regulates the phosphorylation and activation of AKT and mTOR proteins. However, several studies have confirmed the represented results (PI3Kα, MMP9, MMP14, and IL‐6) at the levels of gene expression and total, and activated forms of proteins.^59^, ^60^, ^61^, ^62^, ^63^, ^64^, ^65^, ^66^, ^67^, ^68^, ^69^, ^70^ It is well established that the binding VEGF to its main receptor (VEGFR2) also plays a fundamental role in the differentiation of hematopoietic stem cells, mouse multipotent adult progenitor cells, cardiac stem cells or endothelial progenitor cells to the endothelial cells. Differentiated endothelial cells employ CD31 or von Willebrand Factor (vWF) as specific markers for an endothelial phenotype.^71^, ^72^, ^73^, ^74^, ^75^, ^76^, ^77^, ^78^, ^79^, ^80^, ^81^, ^82^, ^83^, ^84^ It has been shown that PI3K/Akt/mTOR signalling plays key role in the proliferation and differentiation of the endothelial cells. According to the results of this study, Aflibercept (by the VEGF neutralization) down‐regulates PI3K/Akt/mTOR signalling pathway, reduces the proliferation of endothelial cells through the G1 cell cycle arrest and increases their differentiation potential by regulation of the ETS family transcription factors.^85^, ^86^, ^87^ Interleukin 6 is a pleiotropic cytokine, which is produced by different cell types such as endothelial cells. Recent studies have revealed that the expression of IL‐6 is increased in angiogenic process. The IL‑6/JAK/STAT3 signalling pathway has a fundamental role in the angiogenic process, and binding of VEGFA to VEGFR2 leads to STAT3 phosphorylation. On the other hand, activated STAT3 (p‐STAT3) triggers expression of VEGF, binds to the IL‐6 promoter, generates a positive feedback loop, and finally leads to increased level of IL‐6 expression.^70^ Therefore, neutralization of VEGF with Aflibercept leads to a decrease in the phosphorylation of STAT3 and as shown in the current study reduces IL‐6 expression level 24h after HUVEC treatment by the drug.

The classic signalling pathway of IL‐6 leads to activation of STAT3 and AKT through the PI3K pathway. p‐STAT3 translocates to the nucleus and activates MDM2 expression. Moreover, p‐AKT also leads to an increase in the expression and translocation of cytosolic MDM2 to the nucleus. p53 protein prevents the assembly of transcriptional complex on the MMP14 promoter. Degradation of p53 protein (competitive inhibitor of Sp1) is performed by MDM2 and increases expression of MMP14 via binding Sp1 transcription factor.^88^ Therefore, VEGF neutralization via Aflibercept triggers a set of consecutive processes which culminates to decreased level of p‐STAT3 and IL‐6 expression, reduced expression of the MDM2 gene, increased binding of p53 to the MMP14 promoter region and as shown in this study decreased MMP14 expression level, significantly, 24 hours after treatment of HUVECs.

Recent studies have revealed the presence of positive feedback regulation between VEGFA and MMP9 expression in different kinds of surveys. Binding of VEGFA to VEGFR2 regulates MMP9 expression via the ERK signalling pathway.^89^ Therefore, VEGF neutralization via Aflibercept decreases the function of ERK signalling pathway and as shown in this study leads to reduced expression of MMP9 expression, significantly, 6h after treatment. Data showed Aflibercept simultaneously affected angiogenesis, invasion and inflammation processes and identified the conceivable axes that had been affected by the drug. However, integrating the results of Aflibercept on expression of candidate genes with the current biological network centrality analysis suggested that all of the PI3KA, MMP9, MMP14 and IL‐6 genes were less important in terms of bridging centrality and it seems that Aflibercept has a little effect on the expression of nodes that link clusters or densely connected regions. Several studies have revealed that anti‐VEGF drugs induce activation of alternative angiogenic signalling pathways and promotes resistance to the therapeutic regime. The important nodes in terms of bridging centrality (such as ANG2) play a fundamental role in drug resistance. Therefore, while appreciating the significant anti‐angiogenic effect of Aflibercept in patients with different kinds of pathologic angiogenesis‐related disease, research and development of novel therapeutic systems with the ability of simultaneous neutralization of multiple ligands in alternative angiogenic signalling pathways seems to be an inevitable principal.^90^ As mentioned earlier, the current study aimed to investigate the possible signature of the Aflibercept on the components of the downstream of the VEGFR2‐dependent signalling pathway and possibly mechanistic explanation of the anti‐angiogenic effect of the drug. Therefore, it anticipated that there was no need to perform tests that confirm the anti‐angiogenic function of Aflibercept drug such as tube formation or proliferation assay. These tests are usually applied when the goal is confirming the anti‐angiogenic effect of modified or newly designed molecules. Aflibercept has received the approval of the US Food and Drug Administration (FDA) for the treatment of wet AMD and colorectal cancer. There exist many articles that have already investigated Aflibercept effects on tube formation and proliferation of endothelial cells.^91^, ^92^, ^93^ Researchers in the recent study investigated whether ERBB4 rejuvenates aged MSC and how ERBB4 enhances the therapeutic efficacy of aged MSC in treating myocardial infarction (MI). However, before assessment of the effect of ERBB4‐engineered aged mesenchymal stem cells (ER4‐aged MSC) on the angiogenesis‐related protein (such as AKT, ERK, VEGF and FGF2), its function (unlike Aflibercept) must be proved on the angiogenic process through tube formation or proliferation assay since the introduced drug, ERBB4, was not a well know and approved molecule.^94^ So, they should give more basic information on its biology and function.

MTT assay demonstrated more than 95% viability in vascular endothelial cells during application of the peak serum concentration of Aflibercept (0.45 nmol/L).^47^ It is well established that the Aflibercept does not affect the viability of a variety of ocular cells.^95^ So, there was no need for cell viability assessment at this concentration of Aflibercept.

## CONFLICT OF INTEREST

The authors confirm that there are no conflicts of interest.

## AUTHOR CONTRIBUTIONS

**Hamid Latifi‐Navid:** Data curation (equal); Investigation (equal); Methodology (equal); Project administration (equal); Resources (equal); Software (equal); Validation (equal); Visualization (equal); Writing‐review & editing (equal). **Zahra‐Soheila Soheili:** Methodology (equal); Project administration (equal); Supervision (equal); Writing‐review & editing (equal). **Shahram Samiei:** Methodology (equal); Resources (equal); Supervision (equal); Validation (equal). **Mehdi Sadeghi:** Formal analysis (equal). **Sepideh Taghizadeh:** Investigation (equal). **Ehsan Ranaei Pirmardan :** Methodology (equal). **Hamid Ahmadieh:** Formal analysis (equal).

## Supporting information

Figure S1

Figure S2

Table S1

Table S2

Table S3

Table S4

Table S5

Table S6

Table S7

## Data Availability

The data that support the findings of this study are available from the corresponding author upon reasonable request.
