# Supplementary figures and images for: Network analysis and the impact of Aflibercept on specific mediators of angiogenesis in HUVEC cells
Source: J Cell Mol Med. 2021 Jul 11;25(17):8285–99. doi: 10.1111/jcmm.16778 (PMC8419159; doi:10.1111/jcmm.16778)

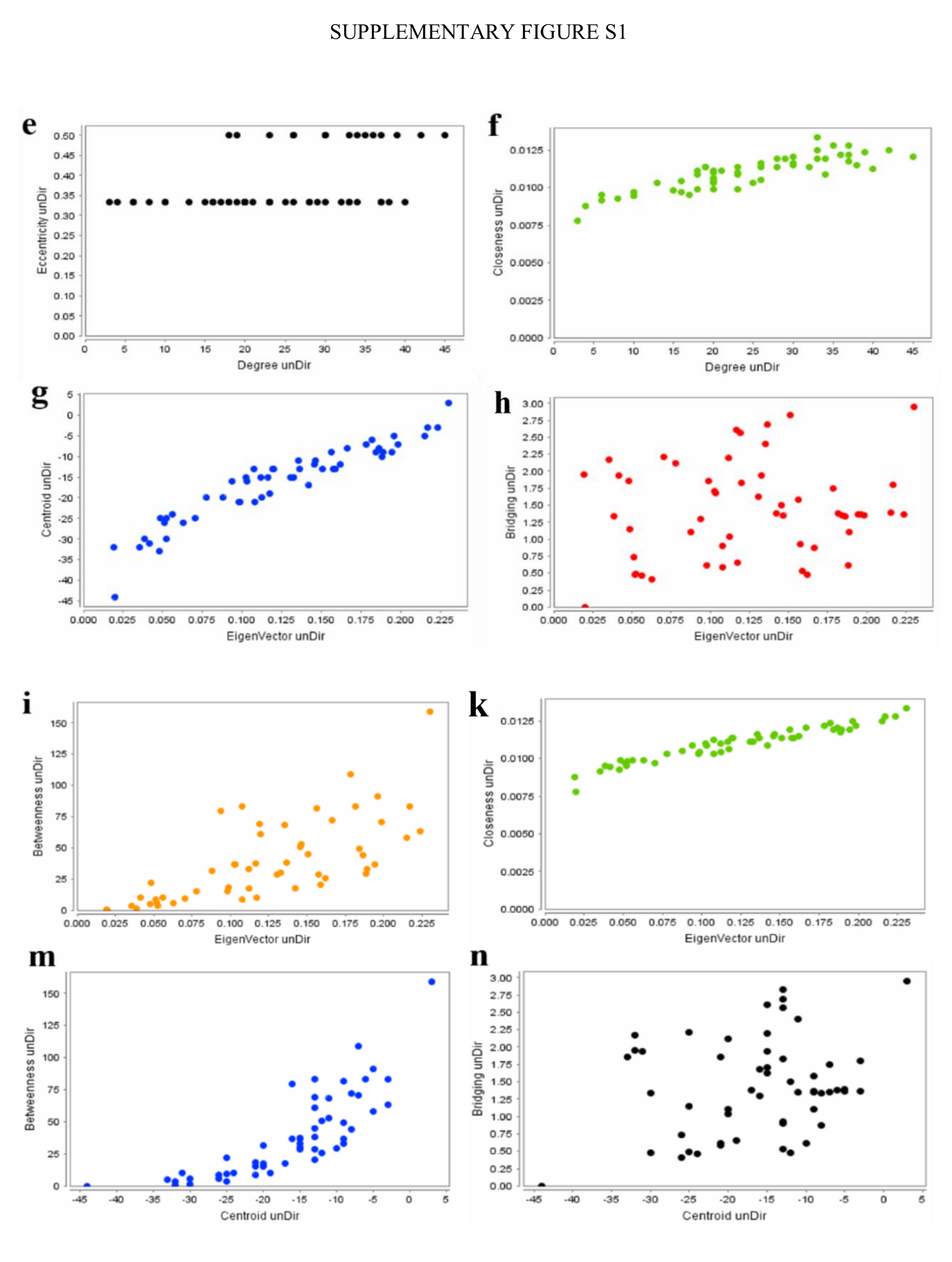

Supplement: Supplementary file 1 — Figure S1 [file JCMM-25-8285-s006.tif]

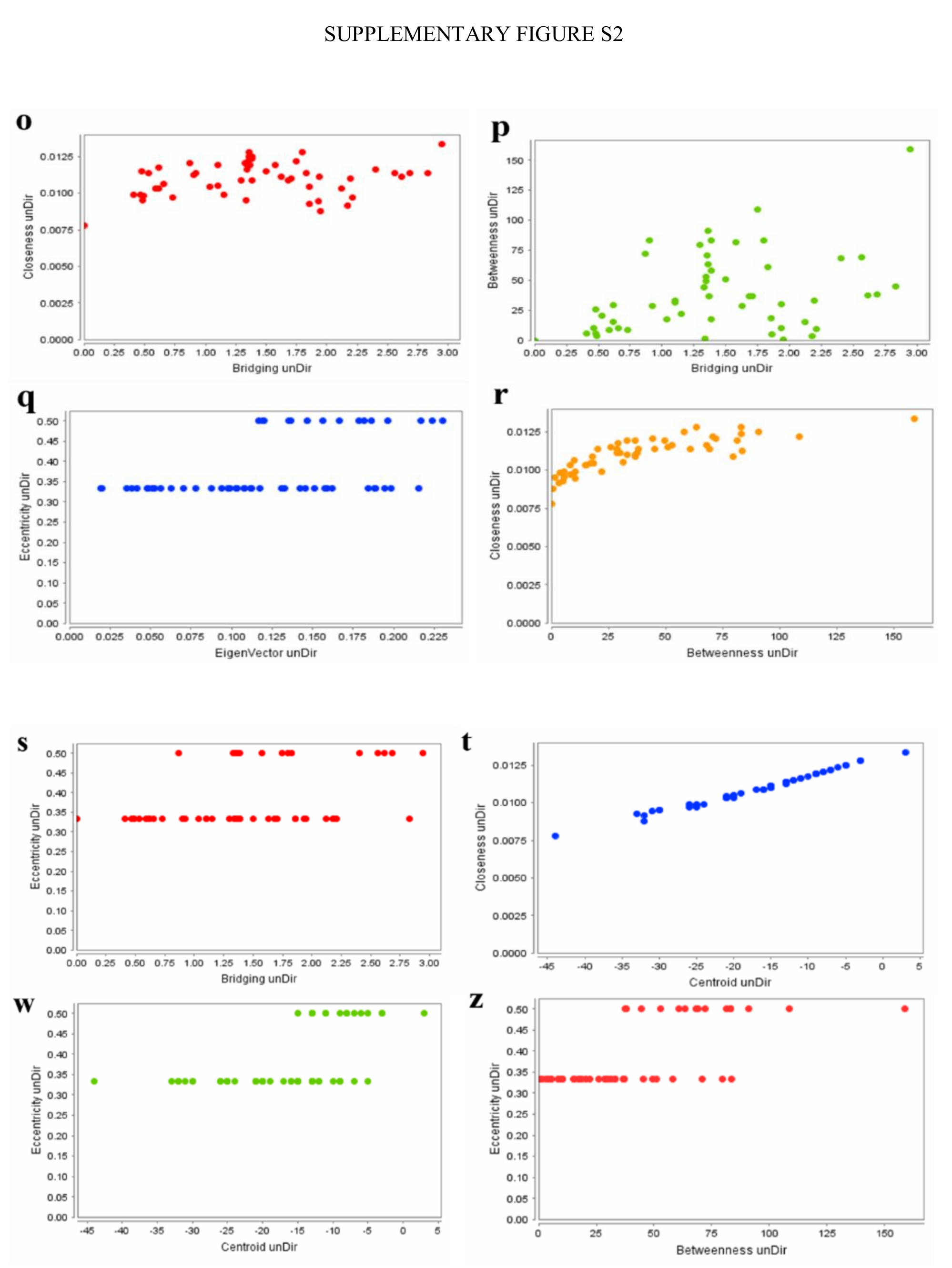

Supplement: Supplementary file 2 — Figure S2 [file JCMM-25-8285-s008.tif]
